# Computational identification and experimental validation of microRNAs binding to the Alzheimer-related gene ADAM10

**DOI:** 10.1186/1471-2350-13-35

**Published:** 2012-05-17

**Authors:** Regina Augustin, Kristina Endres, Sven Reinhardt, Peer-Hendrik Kuhn, Stefan F Lichtenthaler, Jens Hansen, Wolfgang Wurst, Dietrich Trümbach

**Affiliations:** 1 Helmholtz Centre Munich, German Research Centre for Environmental Health (GmbH) and Technical University Munich, Institute of Developmental Genetics, Ingolstädter Landstraße. 1, 85764, Munich-Neuherberg, Germany; 2 Department of Psychiatry and Psychotherapy, University Medical Centre of the Johannes Gutenberg-University Mainz, Untere Zahlbacher Str. 8, 55131, Mainz, Germany; 3 DZNE-German Center for Neurodegenerative Diseases, Schillerstrasse 44, 80336, Munich, Germany; 4 Max Planck Institute of Psychiatry, Kraepelinstr. 2-10, 80804, Munich, Germany

## Abstract

**Background:**

MicroRNAs (miRNAs) are post-transcriptional regulators involved in numerous biological processes including the pathogenesis of Alzheimer’s disease (AD). A key gene of AD, *ADAM10*, controls the proteolytic processing of *APP* and the formation of the amyloid plaques and is known to be regulated by miRNA in hepatic cancer cell lines. To predict miRNAs regulating *ADAM10* expression concerning AD, we developed a computational approach.

**Methods:**

MiRNA binding sites in the human *ADAM10* 3' untranslated region were predicted using the RNA22, RNAhybrid and miRanda programs and ranked by specific selection criteria with respect to AD such as differential regulation in AD patients and tissue-specific expression. Furthermore, target genes of *miR-103*, *miR-107* and *miR-1306* were derived from six publicly available miRNA target site prediction databases. Only target genes predicted in at least four out of six databases in the case of *miR-103* and *miR-107* were compared to genes listed in the AlzGene database including genes possibly involved in AD. In addition, the target genes were used for Gene Ontology analysis and literature mining. Finally, we used a luciferase assay to verify the potential effect of these three miRNAs on *ADAM10* 3'UTR in SH-SY5Y cells.

**Results:**

Eleven miRNAs were selected, which have evolutionary conserved binding sites. Three of them (*miR-103*, *miR-107*, *miR-1306*) were further analysed as they are linked to AD and most strictly conserved between different species. Predicted target genes of *miR-103* (*p*-value = 0.0065) and *miR-107* (*p*-value = 0.0009) showed significant overlap with the AlzGene database except for *miR-1306*. Interactions between *miR-103* and *miR-107* to genes were revealed playing a role in processes leading to AD. *ADAM10* expression in the reporter assay was reduced by *miR-1306* (28%), *miR-103* (45%) and *miR-107* (52%).

**Conclusions:**

Our approach shows the requirement of incorporating specific, disease-associated selection criteria into the prediction process to reduce the amount of false positive predictions. In summary, our method identified three miRNAs strongly suggested to be involved in AD, which possibly regulate *ADAM10* expression and hence offer possibilities for the development of therapeutic treatments of AD.

## Background

MicroRNAs (miRNAs) are on average 22 nucleotides long and play a pivotal role in gene regulation. These small RNAs regulate the gene expression post-transcriptionally by suppression of mRNA translation, stimulation of mRNA deadenylation and degradation or induction of target mRNA cleavage, but have also the potential to activate translation [1,2]. Over half of the mammalian protein coding-genes are regulated by miRNAs and most human mRNAs have binding sites for miRNAs [3]. The interaction of miRNA and target mRNA requires base pairing between the seed sequence (positions 2–8) of the miRNA at the 5^′^ end and a sequence most frequently found in the 3^′^ untranslated region (UTR) of the target mRNA [4]. MiRNAs are involved in neuronal functions like neurite outgrowth and brain development. They were recently described to play a role in human neurodegenerative diseases. Changes in miRNA expression profiles or miRNA target sequences could contribute to the development of Parkinson’s disease and Alzheimer’s disease (AD) [5,6].

Characteristics of AD are insoluble plaques of amyloid β (Aβ) peptides emerging from the cleavage of the amyloid beta precursor protein (*APP*) and neuro-fibrillary tangles in the brains of AD patients [7,8]. The alpha-secretase “a disintegrin and metalloproteinase 10” (*ADAM10*) [9-11] generates soluble secreted amyloid precursor protein-alpha (sAPPα) and avoids formation of plaques, because it cleaves *APP* inside the Aβ sequence [12].

Numerous available computational methods predict a large number of genes targeted by miRNAs regulating gene expression, but only few have been validated experimentally. Many computational predictions are false positives and therefore have to be filtered out [13]. The requirement of target-site conservation in different species including far related species would be a potential way to reduce the false positive rate [14].

In this study we established an approach to identify miRNAs regulating *ADAM10* expression which therefore might influence the progression of AD. The three programs RNA22, RNAhybrid and miRanda predicted potential miRNA binding sites to *ADAM10*. We sought to identify the most interesting miRNAs possibly binding to *ADAM10* with additional selection criteria in particular whether they play a role in AD. Additionally, the most interesting miRNAs were experimentally verified by a luciferase assay. Our results show that *miR-103*, *miR-107* and *miR-1306* influence the expression of *ADAM10* at least in the reporter assay system. These miRNAs could play a role in AD and therefore are interesting candidates to be further analysed concerning their biological function and relation to AD.

## Methods

### miRNA target site prediction databases

MiRNA binding sites to target genes were downloaded from seven different databases: miRBase, 5-Nov-2007, http://www.mirbase.org/[15]; microRNA, September 2008 Release, http://www.microrna.org/microrna/home.do[16]; PicTar via UCSC Table Browser, assembly = May 2004 (NCBI35/hg17), group = Regulation, track = PicTar miRNA, http://genome.ucsc.edu/[17]; PITA, version 6 (31-Aug-2008), http://genie.weizmann.ac.il/pubs/mir07/index.html[18]; RNA22, March 2007, http://cbcsrv.watson.ibm.com/rna22.html[19]; TarBase, June 2008, http://diana.cslab.ece.ntua.gr/tarbase/[20]; TargetScan, Release 5, http://www.targetscan.org/[3]. We established a workflow considering all miRNA target site predictions downloaded.

### miRNA target prediction

We used three prediction programs RNA22, RNAhybrid, miRanda and predicted all binding sites of the miRNA sequences to the 3'UTR sequence of human *ADAM10*.

RNA22 is a pattern-based method for the identification of miRNA-target sites. The method has high sensitivity, is resilient to noise, can be applied to the analysis of any genome without requiring genome-specific retraining and does not rely upon cross-species conservation. Focusing on novel features of miRNA-mRNA interaction RNA22 first finds putative miRNA binding sites in the sequence of interest then identifies the targeting miRNA and hence allows to identify sites targeted by yet-undiscovered miRNAs. An implementation of RNA22 (19-May-2008) is available online at http://cbcsrv.watson.ibm.com/rna22.html[19,21].

The second program RNAhybrid is an extension of the classical RNA secondary structure prediction algorithm from Zuker and Stiegler [22]. It finds the energetically most favorable hybridization sites of a small RNA in a large RNA incorporating ‘seed-match speed-up’, which first searches for seed matches in the candidate targets and only upon finding such matches the complete hybridization around the seed-match is calculated. The user can define the position and length of the seed region with the option to allow for G:U wobble base pairs in the seed pairing. Intramolecular base pairings and branching structures are forbidden and statistical significance of predicted targets is assessed with an extreme value statistics of length normalized minimum free energies, a Poisson approximation of multiple binding sites, and the calculation of effective numbers of orthologous targets in comparative studies of multiple organisms. RNAhybrid, Version 2.1, is available online at http://bibiserv.techfak.uni-bielefeld.de/rnahybrid/[23,24].

The miRanda algorithm is similar to the Smith-Waterman algorithm, but scores based on the complementarity of nucleotides (A = U or G ≡ C) and one G:U wobble pair is allowed in the seed region but has to be compensated by matches in the 3′ end of miRNA. In order to estimate the thermodynamic properties of a predicted pairing between miRNA and 3′UTR sequence, the algorithm uses folding routines from the Vienna 1.3 RNA secondary structure programming library (RNAlib) [25]. A conservation filter is used and optionally some rudimentary statistics about each target site can be generated. MiRanda, September 2008 Release, is available online at http://www.microrna.org/microrna/home.do[21,26].

The parameter setting for RNA22 is: maximum number of “UN-paired” bases within the extent of the seed = 0, extent of seed in nucleotides = 6, minimum number of paired-up bases that you want to see in any reported heteroduplex = 14, maximum value for the folding energy in any reported heteroduplex = −25 kcal/mol. The parameter setting for RNAhybrid is: “-s 3utr_human” (“-s” tells RNAhybrid to quickly estimate statistical parameters from “minimal duplex energies” under the assumption that the target sequences are human 3′UTR sequences). The parameter setting for miRanda is the default parameter setting: gap open penalty = −8, gap extend = −2, score threshold = 50, energy threshold = −20 kcal/mol, scaling parameter = 4.

We retrieved the 3′UTR sequence of *ADAM10* (human *ADAM10* 3′UTR based on transcript NM_001110 (chr15:58888510–58889745)) from NCBI http://www.ncbi.nlm.nih.gov/. We downloaded 703 mature miRNA sequences for *Homo sapiens* from miRBase, version 13.0 http://www.mirbase.org/[15].

### Extraction of best miRNA predictions

The extraction of miRNAs was applied according to the following selection criteria. We checked for each miRNA how many programs predicted the miRNA to bind to human *ADAM10* 3′UTR. The regulation of miRNAs in AD was verified by the publication of Cogswell et al. [27], which provides a list of miRNAs expressed in the tissues hippocampus, cerebellum and medial frontal gyrus. Another possibility to check the expression of miRNAs in the brain is the Mouse Genome Informatics (MGI) database (Mouse Genome Database, The Jackson Laboratory, Bar Harbor, Maine; http://www.informatics.jax.org/) [28]. Literature search by PubMed was done as an additional approval, to search for already described target genes of the miRNAs, especially for target genes involved in AD. Mouse ADAM10 3′UTR based on transcript NM_007399 (chr9:70625902–70628036) from NCBI http://www.ncbi.nlm.nih.gov/ was used for binding site search of mouse miRNAs from miRBase, version 13.0 http://www.mirbase.org/[15]. The parameter setting for RNA22 and miRanda is the same as for human miRNA binding site prediction at the human ADAM10 3′UTR. The parameter setting for RNAhybrid is “-d 1.9,0.28” (1.9 is the location parameter and 0.28 the shape parameter of the assumed extreme value distribution). Additionally, we searched by TargetScan database http://www.targetscan.org/[3] and microRNA database http://www.microrna.org/microrna/home.do[16] for miRNAs binding to human *ADAM10* 3′UTR and compared the TargetScan and microRNA predictions to our list of miRNAs for equal miRNAs. We identified the number of binding sites of a miRNA in the human *ADAM10* 3′UTR predicted by each program. *ADAM10* 3′UTR sequences from ten different species were analysed for conserved regions. The following sequences where taken: human *ADAM10* 3′UTR from transcript NM_001110 (chr15:58888510–58889745), mouse *ADAM10* 3′UTR from transcript NM_007399 (chr9:70625902–70628036), horse *ADAM10* 3′UTR from transcript XM_001498169.1 (chr1:132875124–132876868), dog *ADAM10* 3′UTR from transcript XM_858910 (chr30:26596273–26598436), chimp *ADAM10* 3′UTR from transcript XM_001172393.1 (chr15:55942343–55944774), chicken *ADAM10* 3′UTR from transcript ENSGALT00000034458 (chr10:7949768–7951846), rhesus monkey *ADAM10* 3′UTR from transcript XM_001096908 (chr7:36929437–36932008), zebra fish *ADAM10* 3′UTR from transcript NM_001159314 (chr7:31745579–31747655), opossum *ADAM10* 3′UTR from transcript ENSMODT00000011088 (chr1:162230000–162230183), zebra finch *ADAM10* 3′UTR from transcript XR_054746 (chr10:6638729–6639273). For multiple sequence alignment of the ten *ADAM10* 3′UTR sequences we applied ClustalW Version 2.1 from the European Bioinformatics Institute (EBI) http://www.ebi.ac.uk/[29,30]. We used default parameters except: DNA Weight Matrix = ‘ClustalW’, Clustering = ‘UPGMA’. After extraction of the conserved regions between at least seven species we looked for miRNA binding sites localized in these conserved regions. Additionally, we determined the conservation (given in percentage) of the miRNA binding site sequence from human to each species.

### Statistical analysis

Statistical analysis was performed with R statistical software (R 2.8.0, http://www.r-project.org/). The *p*-value was computed by the R function fisher.test with default settings. The Fisher’s exact test is used to examine the significance of the association (contingency) between the two kinds of classification. Significantly regulated genes were considered, if the *p*-value is equal or below 0.05. We generated Venn diagrams to see the overlap between target genes of *miR-103* and *miR-107* common in 4 out of 6 databases as well as genes in the AlzGene database (http://www.alzgene.org/; Version: 20.06.2011) [31]. Each set of target genes of *miR-103* and *miR-107* common in 4 out of 6 databases as well as the set of target genes of *miR-1306* in the database PITA was explored for enrichment in Gene Ontology [32] by the software Pathway Studio 8.0 (Ariadne Genomics) based on database ResNet 8.0.

### Literature mining and pathway analysis

Literature search by PubMed was done to extract information about the target genes of the miRNAs resulting from Pathway Studio analysis and their relation to AD. To verify the miRNAs searches were performed for miRNA interactions in all PubMed abstracts with the help of the text mining program Pathway Studio 8.0 (Ariadne Genomics) based on the Natural Language Processing (NLP) Technology. Pathway analysis was done with the software Ingenuity Systems IPA 9.0 (http://www.ingenuity.com/) especially with the Path Designer.

### Material

Mature miRNAs and the inactive negative control were from Invitrogen (No. PM11012, PM13206, PM10632, PM10056). All RNA species were dissolved to 5 pmol/μl in nuclease-free water upon arrival, aliquoted and stored at −20°C.

### Cloning of the ADAM10 3′UTR luciferase reporter construct

The 3′UTR of human *ADAM10* was amplified from THP-1 chromosomal DNA using the FailSafe PCR kit (Epicentre) and the following primers:

 AD10_3UTR_for 5′GCGGCCGCGCCCATTCAGCAACCCCAG 3′

 AD10_3UTR_rev 5′GCGGCCGCCACTTGTGCCCGTAGCAGCC 3′.

The obtained DNA fragment was verified by restriction digestion and sequencing. The 3′UTR was subsequently cloned into the *Not*I site of the pCMV-GLuc vector (NEB), which allows to monitor regulated Gaussia luciferase expression in the cell supernatant.

### Cell culture

SH-SY5Y cells were cultivated in phenol red-free DMEM/F12, supplemented with 10% FCS and 1% glutamine at 37°C, 95% air moisture, 5% CO_2_ and passaged twice a week with a splitting rate of ½ to ¼.

### 3′UTR luciferase reporter assay

Retro-transfection was performed using 0.005 μl Lipofectamine 2000 (Invitrogen) per μl OptiMEM-medium and 0.1 pmol/μl miRNA (Invitrogen) or negative control. For combination of miRNA 1306 together with miRNA 103 or 107 a concentration of 0.05 pmol/μl each was used. 2 ng/μl endotoxin-free plasmid DNA of the 3′UTR-reporter vector were added to 45.000 cells per well in 96 well format. Control cells were mock-treated with nuclease-free water instead of RNA molecules.

5 hrs after transfection, the cell supernatant was exchanged to 200 μl culture medium per well. In a preliminary experiment, 10 μl cell supernatant were collected at various time points over a 72 hour period; 48 hours were determined to be the optimal incubation time (data not shown). Therefore, 10 μl cell supernatant were aspirated 48 hours after transfection and stored at −20°C until samples were measured. Secreted Gaussia luciferase was quantitatively analyzed (Renilla-Luciferase assay, Promega) using the FluostarOptima luminometer (BMG). Cell densities were checked by quantitation of protein content in the cell lysate by NanoQuant assay (Roth).

## Results and discussion

### MiRNA prediction

**Figure 1:**
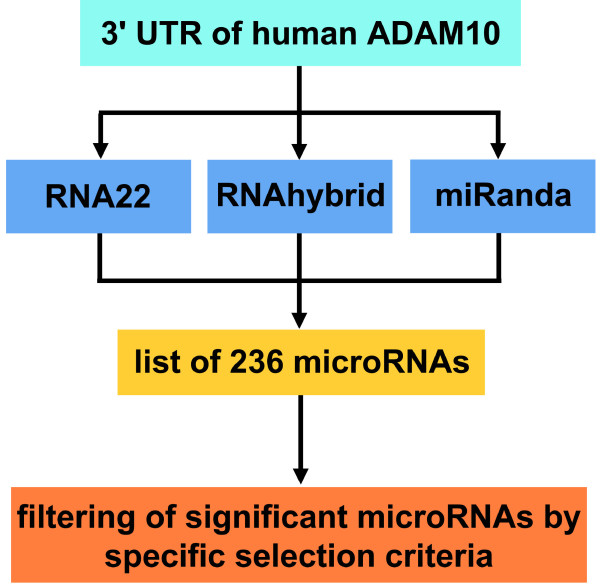
**Computational prediction of miRNAs binding to ADAM10 3'UTR.** Three programs RNA22, RNAhybrid and miRanda are used for the prediction of miRNAs binding to the human *ADAM10* 3'UTR. After retrieving a list of 236 miRNAs by RNA22, RNAhybrid or miRanda we extracted the relevant miRNAs according to selection criteria: prediction by at least two programs, differential regulation in AD patients (Cogswell et al. 2008), tissue-specific expression (MGI), binding to AD key genes, corresponding mouse miRNA binding to the mouse ADAM10 3'UTR sequence predicted by at least two of the three programs, additional prediction by TargetScan and microRNA, occurrence of multiple binding sites and evolutionary conservation.

We established a workflow (Figure 1) to obtain miRNAs possibly binding to the human *ADAM10* 3′UTR. Instead of looking up miRNA target sites of *ADAM10* in the miRNA target site prediction databases, we predicted the binding sites of human miRNAs to human *ADAM10* 3′UTR by the three programs, RNA22, RNAhybrid and miRanda, with the aim to yield a more accurate miRNA target site prediction. Due to the fact that the three prediction programs focus on different aspects for miRNA target site prediction (pattern-based search, seed matching, conservation, energy or structure) various properties of the target sequence are covered (see Methods: “miRNA target prediction”).

122 miRNAs are predicted by at least two programs to bind to human *ADAM10* 3′UTR sequence and 52 of them are significant according to expression and selection criteria described in the following. To consider different aspects of the distinct prediction algorithms at least two programs should predict a miRNA binding site. Important is also the expression of the miRNA in brain provided by the MGI database or the regulation of the miRNA in AD as described by Cogswell and colleagues (2008) in the tissues hippocampus, cerebellum and medial frontal gyrus [27]. An additional confirmation of miRNA being involved in AD is a binding site to a target gene, which is involved in AD, described in the literature and thus the miRNA might regulate also other AD key genes such as ADAM10. Furthermore, the miRNA prediction is strengthened by the corresponding mouse miRNA binding to the mouse ADAM10 3′UTR sequence predicted by at least two of the three miRNA prediction programs RNA22, RNAhybrid and miRanda. Prediction of the miRNA by other webtools such as TargetScan and microRNA is also a confirmation of the miRNA. Multiple binding sites of a single miRNA in the 3′UTR verify the prediction [33].

**Figure 2:**
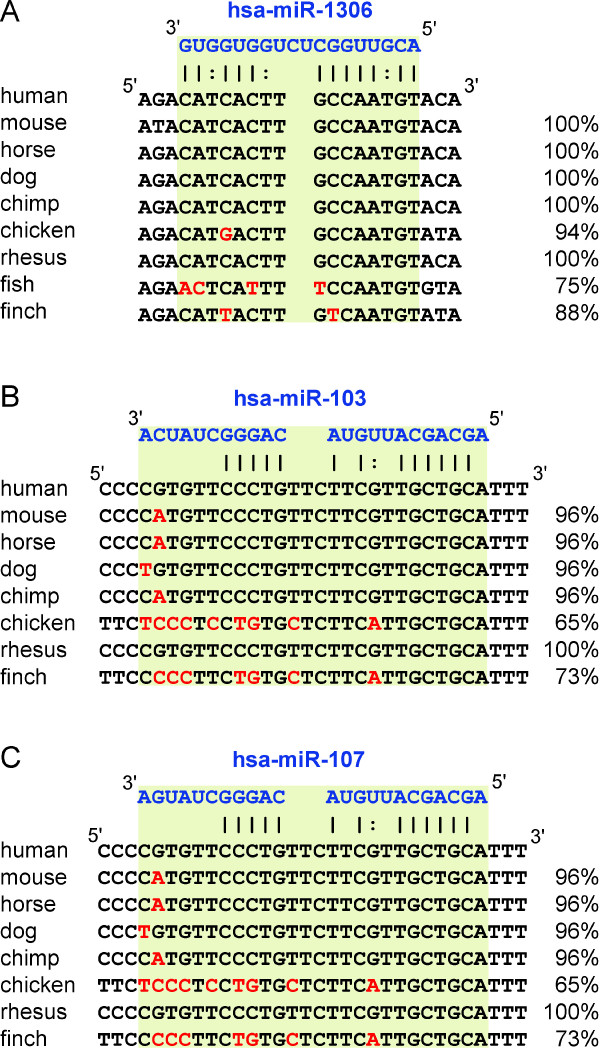
**Conservation of the three miRNA binding sites within the ADAM10 3′UTR.** The figure shows the conservation of the *miR-1306* (**A**), *miR-103* (**B**) and *miR-107* (**C**) binding region (light green) between different species. The blue sequence represents the miRNA binding to the DNA of different species as listed on the left side. On the right side the conservation of the miRNA binding region (light green) from different species to human sequence is given. Nucleotide mismatches in the binding region to human binding region are marked in red. The lines and colons below the miRNA sequence show perfect nucleotide matches and G:U/T wobble pairs, respectively.

**Figure 3:**
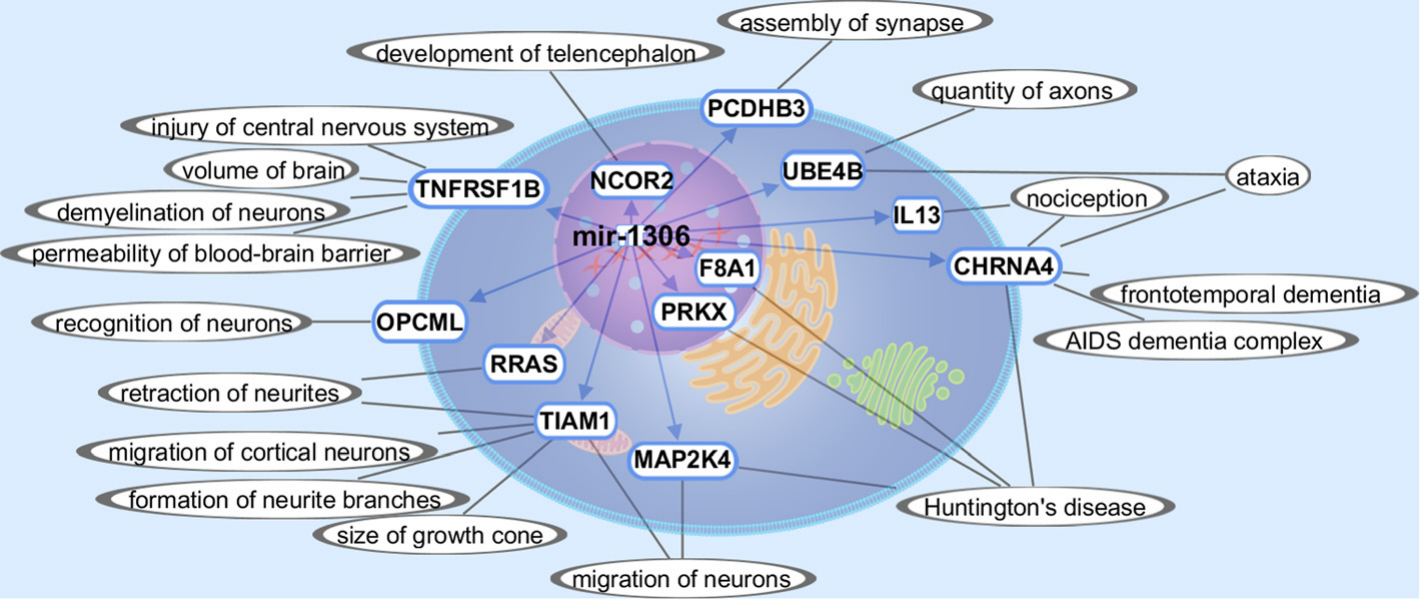
**Target gene predictions for miR-1306.** Twelve target genes of *miR-1306* were predicted by TargetScan concerning their function in AD, brain, nervous system or other neurodegenerative diseases and graphically interrelated by Path Designer (Ingenuity). The miRNA is located in the nucleus and the predicted target genes indicated by blue arrows and blue framed ellipses are located according to their Gene Ontology either in the nucleus, cytoplasm or membrane. The functions of the target genes are denoted by grey lines and grey framed ellipses and derived from Gene Ontology or Ingenuity Expert Findings, which are substantiated by literature.

The list of 52 miRNAs incorporates not conserved as well as conserved miRNAs or rather miRNA binding sites. Filtering according to conservation helps to reduce the number of miRNAs and improves the selection of candidates for further experimental validations. Consideration of conservation across species, including those not developing AD, was chosen as a filter criterion because a correlation between miRNA conservation and disease susceptibility has in general been suggested by Lu et al. [34]. Our selection procedure therefore excludes non-conserved miRNA binding sites, which also might be relevant for development of the disease but are not lost by being included in the list of 52 miRNAs. In regard to AD being a human disease, future analysis of non-conserved miRNAs might also represent a valuable approach which we did not follow in the context of this manuscript. In our analysis eleven miRNA binding sites are conserved across at least seven species, at which four of these miRNAs are also conserved in the far related species zebra fish. The only miRNA binding site *miR-1306* in the human *ADAM10* 3′UTR predicted by all three programs is also conserved in the far related zebra fish as well as in mouse, horse, dog, chimp, chicken, rhesus monkey and zebra finch (Figure 2A). This binding site is located on chromosome 15 positions 58889309–58889324 and the programs RNA22, RNAhybrid and miRanda predicted the binding energy −32.29, -25.7 and −22.55 kcal/mol, respectively. The conservation of the miRNA binding site sequence between human and the species mouse, horse, dog, chimp as well as rhesus monkey is 100%. The conservation of chicken, zebra finch and zebra fish to human in this binding region is 94%, 88% and 75%, respectively. The second most interesting miRNAs possibly binding to human *ADAM10* 3′UTR are *miR-103* as well as *miR-107* both having the same binding site located on chromosome 15 positions 58889443–58889468. This site is predicted by the two programs RNAhybrid and miRanda with binding energy −27.9 and −23.66 kcal/mol for *miR-103*, respectively, as well as −26.2 and −22.28 kcal/mol for *miR-107*, respectively. The conservation of the miRNA binding site sequence between human and the species mouse, horse, dog and chimp is 96%, while the conservation of chicken, rhesus monkey and finch to human is 65%, 100% and 73%, respectively (Figure 2B–C). Additionally, *miR-202miR-423-5pmiR-503miR-184* and *miR-922* bind also to the conserved binding region chromosome 15 positions 58889443–5889473 and *miR-330-5p* (chr15:58889149–58889178), *miR-671-5p* (chr15:58889720–58889745) and *miR-432* (chr15:58889688–58889718) bind to a region with good conservation also to the far related species zebra fish, but these eight miRNAs have no indication to be involved in AD (Table 1). Table 1 shows a ranked list of the best miRNA binding site predictions according to the specific selection criteria. We chose the three most interesting miRNAs 1306, 107 and 103 and performed analyses with the AlzGene database, further miRNA target site prediction databases, Gene Ontology, literature mining and validation experiments to identify the involvement in AD. *MiR-107* and *miR-103* are downregulated with age [35] as well as in AD gray matter [36] and repress the translation of cofilin. In brains of a transgenic mouse model of AD the level of *miR-103* and *miR-107* is decreased while the cofilin protein level is increased which results in the formation of rod-like structures [37]. Furthermore, *miR-107* expression is decreased even in the earliest stages of AD. As *miR-107* regulates beta-site APP-cleaving enzyme 1 (*BACE1*) it might be involved in accelerated disease progression [38]. The downregulation of miR-103 and miR-107 with age could concern a protective effect against plaque formation because reduced levels of these miRNAs would lead to an increased level of the predicted target ADAM10 and its neuroprotective product sAPPα in brains of AD patients. Our observations of strong inhibition (> 40%) of ADAM10 expression in the reporter assay upon application of miR-103 and miR-107 would coincide with such a possible protective influence on amyloid pathology (see “Experimental validation of bioinformatically predicted miRNAs”). According to the publication from Cogswell et al. [27]*miR-103* is differentially expressed in hippocampus and cerebellum in AD. In addition, the program TargetScan verifies the same binding site of *miR-103* and *miR-107* to human *ADAM10*. *MiR-1306* is further analysed due to its good conservation to the far related species zebra fish with only one mismatch in the seed region. It is the only miRNA whose binding site to human *ADAM10* is predicted by all three programs RNA22, RNAhybrid and miRanda, which strengthens the assumption that this binding site is functionally active. Additionally as shown in Figure 3 the hypothesis is verified that *miR-1306* is associated to AD: Twelve predicted target genes of *miR-1306* are involved in processes and functions playing a role in AD, the nervous system and other neurodegenerative diseases. The predictions rely on TargetScan while the dedicated functions are Ingenuity Expert Findings or from Gene Ontology. *MiR-1306* possibly regulates genes like the cholinergic receptor, nicotinic, alpha 4 (*CHRNA4*), tumor necrosis factor receptor superfamily member 1B (*TNFRSF1B*) and mitogen-activated protein kinase kinase 4 (*MAP2K4*), which are associated to AD by the functions frontotemporal dementia, demyelination of neurons and Huntington’s disease, respectively. *MAP2K4* has been found to be involved in AD and is putatively regulated by modules of transcription factor binding sites [39]. Furthermore, miR-1306 is located on chromosome 22 within the second exon of DiGeorge syndrome critical region gene 8 (DGCR8), which is essential for miRNA biogenesis by being a subunit of the microprocessor complex [40]. Evers et al. presents a case of a DiGeorge syndrome patient with the typical deletion in chromosome band 22q11.2, which contains DGCR8, suffering from dementia [41]. 

**Table 1:** List of predicted miRNAs binding to a conserved region of human ADAM10 3'UTR

**miRNA**	**∅ kcal/mol**	**Confirmations**	**Differential regulated in AD**	**Conservation zebra fish**
1306	−26.85	predicted by 3 programs, mouse ADAM10		+
107	−24.24	targets BACE1, predicted by TargetScan, literature for AD, mouse ADAM10		
103	−25.78	predicted by TargetScan, literature for AD, mouse ADAM10	hippocampus, cerebellum	
330-5p	−27.20	predicted by microRNA, mouse ADAM10	hippocampus	+
432	−22.81	predicted by microRNA	cerebellum	+
423-5p	−22.1	mouse ADAM10	hippocampus, medial frontal gyrus	
671-5p	−27.61	mouse ADAM10		+
922	−27.99	predicted by microRNA		
503	−25.41	predicted by microRNA, mouse ADAM10		
202	−25.33	predicted by microRNA, mouse ADAM10		
184	−23.33	mouse ADAM10		

The best eleven predicted miRNAs, which bind to a conserved region of human *ADAM10* 3'UTR, are shown with additional information like the average predicted binding energy in kcal/mol (column 2), verifications of the miRNAs from literature, by additional mouse ADAM10 prediction or other prediction programs (column 3), the regulation of miRNAs in AD according to Cogswell et al. (column 4) and the conservation of the binding region in zebra fish marked with + in the last column.

### Prediction of miRNA target genes and their relation to AD

**Figure 4:**
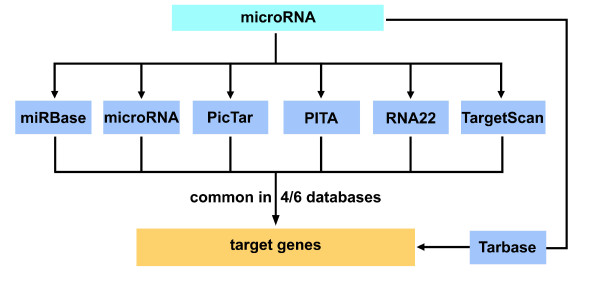
**Workflow for miRNA target site prediction.** Seven databases miRBase, microRNA, PicTar, PITA, RNA22, TargetScan and Tarbase, which is experimentally supported by miRNA targets, are incorporated in the prediction of miRNA target sites. The input is a miRNA and the output is a set of target genes common to four out of six databases. Tarbase contains experimentally supported miRNA targets and not in-silico predicted miRNA binding sites: all miRNA target genes from Tarbase are automatically included in the output of the analysis.

**Figure 5:**
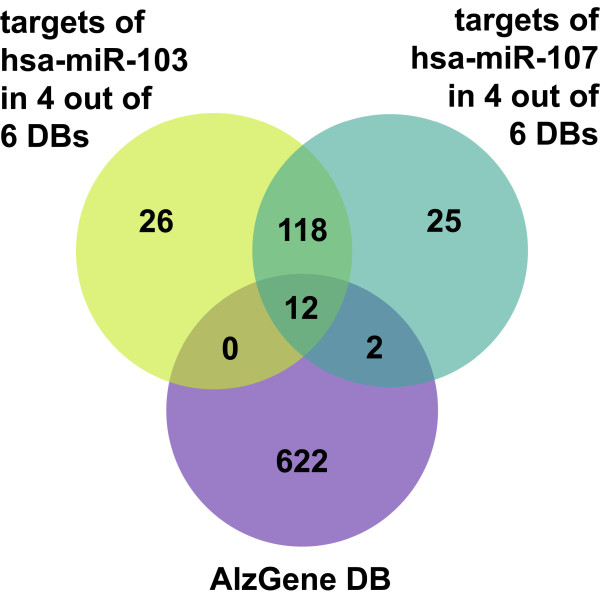
**Venn Diagram.** The Venn diagram shows the significant overlap of the target genes of *miR-103* and *miR-107* common in four out of six databases as well as the genes of AlzGene database.

To confirm the relationship of the three miRNAs to AD we searched for target gene predictions of these miRNAs in the six databases miRBase, microRNA, PicTar, PITA, RNA22, as well as TargetScan (Figure 4). The combination of different miRNA target site prediction databases and the restriction of the output to four out of six databases in the case of *miR-103* and *miR-107* are important to reduce the amount of false positive miRNA target sites in the end. In the case of *miR-1306* there is no restriction of the output, because only the database PITA incorporates predictions for this miRNA. Additionally, Tarbase can be included in the analysis. The database Tarbase has a special position, because in contrast to the other six databases Tarbase contains experimentally supported miRNA targets and not in-silico predicted miRNA binding sites. All miRNA target sites from Tarbase are automatically included in the output of the analysis. In contrast to the other six databases Tarbase doesn’t contain false positive miRNA target sites. In our case Tarbase doesn’t contain target genes for the miRNAs: *miR-103*, *miR-107* and *miR-1306*. The six miRNA target site prediction databases miRBase, microRNA, PicTar, PITA, RNA22, and TargetScan contain altogether 18915 different (according to EntrezGene) human genes at which PITA alone contains 16819 genes. After the analysis we got 156 and 157 target genes for *miR-103* and *miR-107*, respectively, common in four out of six databases, and 890 target genes for *miR-1306* of database PITA. As we focus on miRNAs playing a role in AD, we used the AlzGene database to see which target genes of the miRNAs have a genetic association with AD. AlzGene database is a regularly updated aggregation of all published genetic association studies including GWAS (genome-wide association studies) performed on AD phenotypes. 636 and 591 genes of AlzGene database overlap with 18915 genes of the six miRNA target site prediction databases and 16819 genes of database PITA, respectively. The overlap between AlzGene database genes and the target genes of *miR-103*, *miR-107* as well as *miR-1306* are 12, 14 (Figure 5) and 24 genes, respectively (Additional file 1). *MiR-103* and *miR-107* have 130 target genes in common (Figure 5, Additional file 1). Applying a Fisher’s exact test we got a *p*-value of 0.0065, 0.0009 and 0.1904 for the overlap of *miR-103*, *miR-107* and *miR-1306*, respectively, with the AlzGene database, which shows that 12 and 14 are significant high numbers of overlapping genes between the target genes and the AlzGene database. This result suggests that *miR-103* and *miR-107* might play a role in AD. It is not remarkable that the *p*-value for the overlap of *miR-1306* with the AlzGene database is not significant as the restriction to four out of six databases was not possible, which consequentially leads to the inclusion of a lot of false positive target genes in the 890 target genes of *miR-1306*.

### Gene ontology

Additionally, we did a Gene Ontology analysis with the predicted target genes of the three miRNAs to validate the functionality of the miRNAs and their involvement to AD. With the help of the literature mining tool Pathway Studio we searched for molecular functions and biological processes common to the target genes of the three miRNAs. The molecular function calcium ion binding is significant in all three miRNA analyses (*miR-103*: *p*-value = 0.0011; *miR-107*: *p*-value = 0.0025; *miR-1306*: *p*-value = 2.1 × 10^−5^) and is considered to be involved in AD. Calcium ions are found in an elevated level in tangle-bearing neurons of AD patients compared to healthier neurons [42]. Further, an abnormal increase of intracellular Calcium ion levels in neurites associated with Aβ deposits was demonstrated in a mouse model of AD [43]. An additional evidence is given by the significant biological processes learning (*miR-103*: *p*-value = 0.0008; *miR-107*: *p*-value = 3.3 × 10^−5^; *miR-1306*: *p*-value = 0.0003), brain development (*miR-103*: *p*-value = 0.0023; *miR-107*: *p*-value = 0.0004; *miR-1306*: *p*-value = 7.1 × 10^−6^) and nervous system development (*miR-103*: *p*-value = 0.0004; *miR-107*: *p*-value = 0.0004; *miR-1306*: *p*-value = 6.9 × 10^−8^) that the three miRNAs are involved in AD. Mouse models with an overexpression of *ADAM10* showed a positive effect of the α-secretase on learning and memory and mice with a dominant negative mutant form of *ADAM10* had learning deficiencies [12]. Environmental influences occurring during brain development predefine the expression and regulation of APP. As a consequence levels of APP and Aβ are increased causing AD later in life [44]. The Aβ fragments forming plaques are of varying length depending on the site of cleavage. The Aβ_42_ fragment is a ligand for the cellular prion protein, which is important for nervous system development [45]. (See Additional files 2,3 and 4: List of enriched target genes of miR-103/107/1306 in Gene Ontology).

### Literature mining

**Figure 6:**
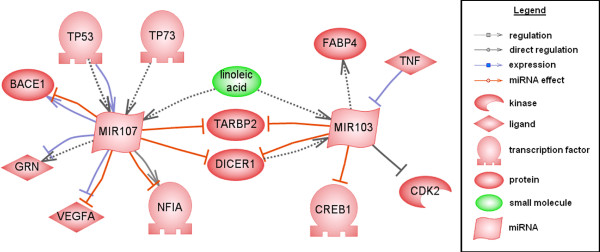
**Interaction network of miR-103 and miR-107.** The network (established by Pathway Studio) shows already published interactions between *miR-103* as well as *miR-107* and genes known to be involved in AD or neurodegenerative processes. The different types of proteins or small molecules are indicated by different symbols and the various interactions like regulation, expression and miRNA effect are also displayed by various arrows.

Furthermore, a network (Figure 6) containing already published interactions of *miR-103* and *miR-107* with genes involved in AD or included in the AlzGene database was established using the literature mining tool Pathway Studio. This network allowed confirming the relation of the two miRNAs to AD by their respective target genes. The genes dicer 1, ribonuclease type III (*Dicer*) and TAR (HIV-1) RNA binding protein 2 (*TARBP2*) targeted by *miRNA-103* and *miRNA-107* are components of the miRNA-processing complex [46]. Besides those two genes, a link between the two miRNAs is provided by linoleic acid [47], which probably affects AD by increasing the expression of Presenilin1 (*PSEN1*) and Aβ [48]. Another target gene in the network, Granulin (*GRN*), is regulated by *miR-107*[49] which regulates *BACE1* as well [38]. Therefore both genes might be involved in neurodegenerative diseases especially AD. The tumor suppressors *TP53* as well as *TP73* appear to regulate the processing of *miR-107*[46]. M*iR-103* increases the expression level of fatty acid binding protein 4 (*FABP4*) while its expression is reduced by tumor necrosis factor (*TNF*) [50]. All six target genes of the miRNAs 103 and 107 *GRNBACE1TP53TP73FABP4* and *TNF* are included in the AlzGene database, hence putatively playing a role in AD. The four remaining target genes cyclin-dependent kinase 2 (*CDK2*), cAMP responsive element binding protein 1 (*CREB1*), nuclear factor I/A (*NFIA*) and vascular endothelial growth factor A (*VEGFA*) of the network are mentioned in literature to be involved directly or in processes developing AD. *miR-103* directly binds and represses *CDK2* and *CREB1* through 3′UTR binding [51]. *CDK2* is a key regulator in neuronal differentiation with the downregulation of *CDK2* as crucial event [52] and neuronal differentiation is regulated by *PSEN1*, a major key gene of AD [53]. The transcription factor *CREB1* is involved in several types of learning and memory. A direct involvement in AD is seen in some mouse models, where its activity is impaired [54]. *NFIA* is negatively regulated by *miR-107*[55] and plays an important role in the formation of the corpus callosum in the developing brain. The disruption of *NFIA* results in agenesis of the corpus callosum [56], whereas the size of the corpus callosum is significantly reduced in AD patients [57]. The expression of the hypoxia-regulated gene *VEGFA* is decreased by *miR-107*[58]. Additionally, it is known, that polymorphisms within the *VEGFA* promoter region are associated with increased risk for AD, by reducing the neuroprotective effect of *VEGFA*[59]. These findings of the literature and AlzGene database confirm the biological role of the target genes in neurodegenerative processes and hence the involvement of *miR-103* and *miR-107* in AD. 

### Experimental validation of bioinformatically predicted miRNAs

**Figure 7:**
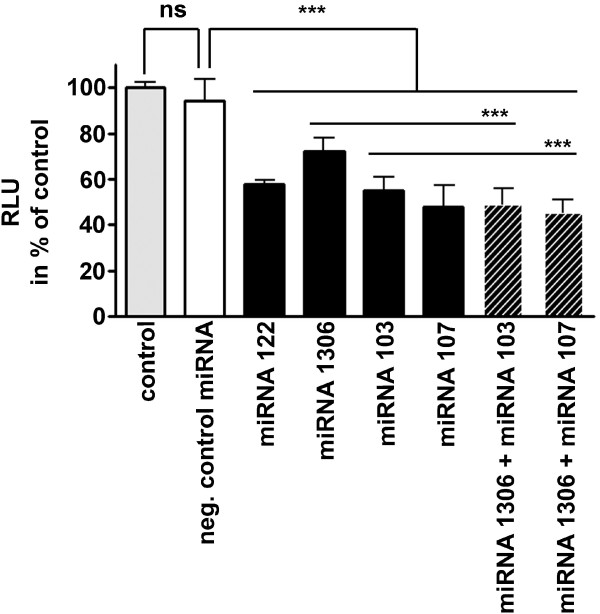
**Experimental validation of selected miRNAs.** SH-SY5Y cells were transiently cotransfected with the Gaussia reporter vector harbouring the 3'UTR of ADAM10 downstream of the luciferase coding sequence together with the respective miRNAs. *MiR-122*, which has been described by Bai et al. [60] to target *ADAM10*, served as a positive control. After 48 hrs of incubation, luminescence was measured in the cell supernatant. Values obtained for control (water) treated cells were set to 100%, data represent mean ± SD of three independent experiments performed in triplicate. *** *P* < 0.001, ns = not significant, RLU = Relative Light Units.

To demonstrate that the selected miRNAs 1306, 103 and 107 directly regulate *ADAM10* expression by interaction with the 3′UTR of the human gene, we performed cotransfection experiments with a Gaussia reporter vector harbouring the 3′UTR of *ADAM10* downstream of the luciferase coding sequence together with the respective miRNAs. As a positive control we used *miR-122* which has been identified and validated as an important regulator of *ADAM10* in hepatocellular carcinoma by an experimental approach [60]. The programs RNA22, RNAhybrid and miRanda predicted a miR-122 binding site to human *ADAM10* 3′UTR with binding energy −31.2, -25.6 and −21.84 kcal/mol (on average: −26.21 kcal/mol), respectively, comparable to the miR-1306 binding site prediction (see Results and Discussion: “miRNA prediction”). Time resolved measurement revealed that 48 hrs incubation period resulted in maximal effects of the miRNAs in SH-SY5Y cells (data not shown): while the negative control miRNA had no impact on luciferase activity measured in the cell supernatant, *miR-122* reduced the luminescent signal to 57% as compared to water treated control cells (Figure 7). This effect is even higher than the one observed in the initial publication from Bai et al. (2009) [60] but might be due to the different reporter enzymes or cell lines used. The three miRNAs identified by bioinformatical approaches and integration of literature mining all showed a significant decreasing effect on the *ADAM10* 3′UTR-reporter construct: *miR-1306* lowered the luminescent signal to 72%, *miR-103* to 55% and *miR-107* to 48% of control. While *miR-103* and *miR-107* target the same DNA sequence within the *ADAM10* 3′UTR (see Figure 2), *miR-1306* has a binding site in closer proximity to the Stop codon. We therefore combined *miR-1306* and *miR-103* or *miR-107*, respectively, but observed no distinct significant synergistic effect (*miR-1306* vs. *miR-1306/103 p* < 0.001; *miR-1306* vs. *miR-1306/107 p* < 0.001). This might be due to the applied concentration which was reduced to 50% in the combinations (see methods “3′UTR luciferase reporter assay”). These experimental results suggest an influence of *miR-103miR-107* and *miR-1306* on *ADAM10* expression. Nevertheless, the biological impact of either miRNA has to be elucidated further, e.g. by mRNA and protein measurements. Assessing the effect of the selected miRNAs on pathological features in AD mouse models would also help to understand their distinct role in pathogenesis. However, this study shows that the computational workflow consisting of prediction programs and specific selection criteria is a suitable tool for the identification of miRNAs influencing key genes of diseases such as Alzheimer’s disease. 

## Conclusions

We established a computational approach for the identification of miRNAs putatively influencing the expression of *ADAM10*. A potential functionality of selected miRNAs 103, 107 and 1306 was confirmed by 3′UTR luciferase reporter assay. These results show that the evolutionary conservation of the target gene binding site facilitates miRNA candidate selection independently from the disease for further experimental validation. Moreover, these experiments underline the reliability of our computational approach, which is a combination of characteristics of the prediction software and specific selection criteria for filtering out false positive predictions: disease relevance, specificity of expression, evolutionary conservation of binding sites and occurrence of multiple binding sites. This workflow can also be applied to key genes of other diseases with adjustment of the selection criteria according to the scientific research interest. Our approach provides a new selection tool for identification and ranking of AD-related miRNAs, but to elucidate a profound pathological role of selected candidates, further experiments have to be done.

## Abbreviations

Chr = Chromosome; Human = Homo sapiens; Mouse = Mus musculus; Horse = Equus caballus; Dog = Canis lupus familiaris; Chimp = Pan troglodytes; Chicken = Gallus gallus; Rhesus monkey = Macaca mulatta; Zebra fish = Danio rerio; Opossum = Monodelphis domestica; Zebra finch = Taeniopygia guttata.

## Competing interests

The authors declare that they have no competing interests.

## Authors’ contributions

RA performed the bioinformatic analyses and wrote the manuscript. RA and DT designed the computational workflow. KE designed the experimental system. SR performed transfection and luciferase reporter assay experiments. SFL and PHK contributed to the planning of the whole project. JH participated in the conception of this work. WW directed the work. DT planed and coordinated the entire project. All authors read and approved the final manuscript.

## Pre-publication history

The pre-publication history for this paper can be accessed here:

http://www.biomedcentral.com/1471-2350/13/35/prepub

## Supplementary Material

Additional file 1 **List of predicted target genes common in 4 out of 6 DBs.** The table shows a list of predicted target genes of miR-103, miR-107, miR-1306. These target genes are either listed in AlzGene DB and target gene of at least one miRNA or target gene of at least two miRNAs (column 3). Beside the gene symbol the Entrez GeneID is given. Click here for file

Additional file 2 **List of enriched target genes of miR-103 in Gene Ontology.** The table shows the Gene Ontology entities with enrichment of predicted target genes of miR-103. Additionally a *p*-value and FDR value is given. Click here for file

Additional file 3 **List of enriched target genes of miR-107 in Gene Ontology.** The table shows the Gene Ontology entities with enrichment of predicted target genes of miR-107. Additionally a *p*-value and FDR value is given. Click here for file

Additional file 4 **List of enriched target genes of miR-1306 in Gene Ontology.** The table shows the Gene Ontology entities with enrichment of predicted target genes of miR-1306. Additionally a *p*-value and FDR value is given. Click here for file

## References

[B1] Chekulaeva M Filipowicz W Mechanisms of miRNA-mediated post-transcriptional regulation in animal cells Curr Opin Cell Biol 2009 21 452 460 10.1016/j.ceb.2009.04.009 19450959

[B2] Vasudevan S Steitz JA AU-rich-element-mediated upregulation of translation by FXR1 and Argonaute 2 Cell 2007 128 1105 1118 10.1016/j.cell.2007.01.038 17382880 PMC3430382

[B3] Friedman RC Farh KK-H Burge CB Bartel DP Most mammalian mRNAs are conserved targets of microRNAs Genome Res 2009 19 92 105 18955434 10.1101/gr.082701.108 PMC2612969

[B4] Fabian MR Sonenberg N Filipowicz W Regulation of mRNA translation and stability by microRNAs Annu Rev Biochem 2010 79 351 379 10.1146/annurev-biochem-060308-103103 20533884

[B5] Hébert SS De Strooper B Alterations of the microRNA network cause neurodegenerative disease Trends Neurosci 2009 32 199 206 10.1016/j.tins.2008.12.003 19268374

[B6] Satoh J MicroRNAs and their therapeutic potential for human diseases: aberrant microRNA expression in Alzheimer’s disease brains J Pharmacol Sci 2010 114 269 275 10.1254/jphs.10R11FM 20953120

[B7] Crews L Rockenstein E Masliah E APP transgenic modeling of Alzheimer’s disease: mechanisms of neurodegeneration and aberrant neurogenesis Brain Struct Funct 2010 214 111 126 10.1007/s00429-009-0232-6 20091183 PMC2847155

[B8] Cole SL Vassar R The role of amyloid precursor protein processing by BACE1, the beta-secretase, in Alzheimer disease pathophysiology J Biol Chem 2008 283 29621 29625 10.1074/jbc.R800015200 18650431 PMC2662048

[B9] Lammich S Kojro E Postina R Gilbert S Pfeiffer R Jasionowski M Haass C Fahrenholz F Constitutive and regulated alpha-secretase cleavage of Alzheimer’s amyloid precursor protein by a disintegrin metalloprotease Proc Natl Acad Sci 1999 96 3922 3927 10.1073/pnas.96.7.3922 10097139 PMC22396

[B10] Kuhn PH Wang H Dislich B Colombo A Zeitschel U Ellwart JW Kremmer E Rossner S Lichtenthaler SF ADAM10 is the physiologically relevant, constitutive alpha-secretase of the amyloid precursor protein in primary neurons EMBO J 2010 29 3020 3032 10.1038/emboj.2010.167 20676056 PMC2944055

[B11] Lichtenthaler SF Haass C Steiner H Regulated intramembrane proteolysis – lessons from amyloid precursor protein processing J Neurochem 2011 117 779 796 10.1111/j.1471-4159.2011.07248.x 21413990

[B12] Postina R Schroeder A Dewachter I Bohl J Schmitt U Kojro E Prinzen C Endres K Hiemke C Blessing M Flamez P Dequenne A Godaux E van Leuven F Fahrenholz F A disintegrin-metalloproteinase prevents amyloid plaque formation and hippocampal defects in an Alzheimer disease mouse model J Clin Invest 2004 113 1456 1464 15146243 10.1172/JCI20864 PMC406531

[B13] Watanabe Y Tomita M Kanai A Computational methods for microRNA target prediction Methods Enzymol 2007 427 65 86 17720479 10.1016/S0076-6879(07)27004-1

[B14] Lewis BP Burge CB Bartel DP Conserved seed pairing, often flanked by adenosines, indicates that thousands of human genes are microRNA targets Cell 2005 120 15 20 10.1016/j.cell.2004.12.035 15652477

[B15] Griffiths-Jones S Saini HK van Dongen S Enright AJ miRBase: tools for microRNA genomics Nucleic Acids Res 2008 36 D154 D158 10.1093/nar/gkn221 17991681 PMC2238936

[B16] Betel D Wilson M Gabow A Marks DS Sander C The microRNA.org resource: targets and expression Nucleic Acids Res 2008 36 D149 D153 18158296 10.1093/nar/gkm995 PMC2238905

[B17] Krek A Grün D Poy MN Wolf R Rosenberg L Epstein EJ MacMenamin P da Piedade I Gunsalus KC Stoffel M Rajewsky N Combinatorial microRNA target predictions Nat Genet 2005 37 495 500 10.1038/ng1536 15806104

[B18] Kertesz M Iovino N Unnerstall U Gaul U Segal E The role of site accessibility in microRNA target recognition Nat Genet 2007 39 1278 1284 10.1038/ng2135 17893677

[B19] Miranda KC Huynh T Tay Y Ang Y-S Tam W-L Thomson AM Lim B Rigoutsos I A pattern-based method for the identification of MicroRNA binding sites and their corresponding heteroduplexes Cell 2006 126 1203 1217 10.1016/j.cell.2006.07.031 16990141

[B20] Papadopoulos GL Reczko M Simossis VA Sethupathy P Hatzigeorgiou AG The database of experimentally supported targets: a functional update of TarBase Nucleic Acids Res 2009 37 D155 D158 10.1093/nar/gkn809 18957447 PMC2686456

[B21] Witkos TM Koscianska E Krzyzosiak WJ Practical Aspects of microRNA Target Prediction Curr Mol Med 2011 11 93 109 10.2174/156652411794859250 21342132 PMC3182075

[B22] Zuker M Stiegler P Optimal computer folding of large RNA sequences using thermodynamics and auxiliary information Nucleic Acids Res 1981 9 133 148 10.1093/nar/9.1.133 6163133 PMC326673

[B23] Rehmsmeier M Steffen P Hochsmann M Giegerich R Fast and effective prediction of microRNA/target duplexes RNA 2004 10 1507 1517 10.1261/rna.5248604 15383676 PMC1370637

[B24] Krüger J Rehmsmeier M RNAhybrid: microRNA target prediction easy, fast and flexible Nucleic Acids Res 2006 34 W451 W454 10.1093/nar/gkl243 16845047 PMC1538877

[B25] Wuchty S Fontana W Hofacker IL Schuster P Complete suboptimal folding of RNA and the stability of secondary structures Biopolymers 1999 49 145 165 10.1002/(SICI)1097-0282(199902)49:2<145::AID-BIP4>3.0.CO;2-G 10070264

[B26] Enright AJ John B Gaul U Tuschl T Sander C Marks DS MicroRNA targets in Drosophila Genome Biol 2003 5 R1 10.1186/gb-2003-5-1-r1 14709173 PMC395733

[B27] Cogswell JP Ward J Taylor IA Waters M Shi Y Cannon B Kelnar K Kemppainen J Brown D Chen C Prinjha RK Richardson JC Saunders AM Roses AD Richards CA Identification of miRNA changes in Alzheimer’s disease brain and CSF yields putative biomarkers and insights into disease pathways J Alzheimers Dis 2008 14 27 41 18525125 10.3233/jad-2008-14103

[B28] Bult CJ Eppig JT Kadin JA Richardson JE Blake JA Mouse Genome Database Group The Mouse Genome Database (MGD): mouse biology and model systems Nucleic Acids Res 2008 36 D724 D728 18158299 10.1093/nar/gkm961 PMC2238849

[B29] Larkin MA Blackshields G Brown NP Chenna R McGettigan PA McWilliam H Valentin F Wallace IM Wilm A Lopez R Thompson JD Gibson TJ Higgins DG Clustal W and Clustal X version 2.0 Bioinformatics 2007 23 2947 2948 10.1093/bioinformatics/btm404 17846036

[B30] Goujon M McWilliam H Li W Valentin F Squizzato S Paern J Lopez R A new bioinformatics analysis tools framework at EMBL-EBI Nucleic Acids Res 2010 38 W695 W699 10.1093/nar/gkq313 20439314 PMC2896090

[B31] Bertram L McQueen MB Mullin K Blacker D Tanzi RE Systematic meta-analyses of Alzheimer disease genetic association studies: the AlzGene database Nat Genet 2007 39 17 23 10.1038/ng1934 17192785

[B32] Ashburner M Ball CA Blake JA Botstein D Butler H Cherry JM Davis AP Dolinski K Dwight SS Eppig JT Harris MA Hill DP Issel-Tarver L Kasarskis A Lewis S Matese JC Richardson JE Ringwald M Rubin GM Sherlock G Gene Ontology: tool for the unification of biology Nat Genet 2000 25 25 29 10.1038/75556 10802651 PMC3037419

[B33] Sethupathy P Megraw M Hatzigeorgiou AG A guide through present computational approaches for the identification of mammalian microRNA targets Nat Meth 2006 3 881 886 10.1038/nmeth954 17060911

[B34] Lu M Zhang Q Deng M Miao J Guo Y Gao W Cui Q An analysis of human microRNA and disease associations PLoS One 2008 3 e3420 10.1371/journal.pone.0003420 18923704 PMC2559869

[B35] Noren Hooten N Abdelmohsen K Gorospe M Ejiogu N Zonderman AB Evans MK microRNA expression patterns reveal differential expression of target genes with age PLoS One 2010 5 e10724 10.1371/journal.pone.0010724 20505758 PMC2873959

[B36] Wang WX Huang Q Hu Y Stromberg AJ Nelson PT Patterns of microRNA expression in normal and early Alzheimer’s disease human temporal cortex: white matter versus gray matter Acta Neuropathol 2011 121 193 205 10.1007/s00401-010-0756-0 20936480 PMC3073518

[B37] Yao J Hennessey T Flynt A Lai E Beal MF Lin MT MicroRNA-related cofilin abnormality in Alzheimer’s disease PLoS One 2010 5 e15546 10.1371/journal.pone.0015546 21179570 PMC3002958

[B38] Wang WX Rajeev BW Stromberg AJ Ren N Tang G Huang Q Rigoutsos I Nelson PT The expression of microRNA miR-107 decreases early in Alzheimer’s disease and may accelerate disease progression through regulation of beta-site amyloid precursor protein-cleaving enzyme 1 J Neurosci 2008 28 1213 1223 10.1523/JNEUROSCI.5065-07.2008 18234899 PMC2837363

[B39] Augustin R Lichtenthaler SF Greeff M Hansen J Wurst W Trümbach D Bioinformatics identification of modules of transcription factor binding sites in Alzheimer’s disease-related genes by in silico promoter analysis and microarrays International Journal of Alzheimer’s Disease 2011 2011 154325 21559189 10.4061/2011/154325 PMC3090009

[B40] Wang Y Medvid R Melton C Jaenisch R Blelloch R DGCR8 is essential for microRNA biogenesis and silencing of embryonic stem cell self-renewal Nat Genet 2007 39 380 385 10.1038/ng1969 17259983 PMC3008549

[B41] Evers LJ Vermaak MP Engelen JJ Curfs LM The velocardiofacial syndrome in older age: dementia and autistic features Genet Couns 2006 17 333 340 17100202

[B42] Nixon RA Saito KI Grynspan F Griffin WR Katayama S Honda T Mohan PS Shea TB Beermann M Calcium-activated neutral proteinase (calpain) system in aging and Alzheimer’s disease Ann N Y Acad Sci 1994 747 77 91 7847693 10.1111/j.1749-6632.1994.tb44402.x

[B43] Mattson MP ER calcium and Alzheimer’s disease: in a state of flux Sci Signal 2010 3 pe10 10.1126/scisignal.3114pe10 20332425 PMC3091478

[B44] Zawia NH Lahiri DK Cardozo-Pelaez F Epigenetics, oxidative stress, and Alzheimer disease Free Radic Biol Med 2009 46 1241 1249 10.1016/j.freeradbiomed.2009.02.006 19245828 PMC2673453

[B45] Kim D Tsai LH Bridging physiology and pathology in AD Cell 2009 137 997 1000 10.1016/j.cell.2009.05.042 19524503

[B46] Boominathan L The tumor suppressors p53, p63, and p73 are regulators of microRNA processing complex PLoS One 2010 5 e10615 10.1371/journal.pone.0010615 20485546 PMC2868896

[B47] Parra P Serra F Palou A Expression of adipose microRNAs is sensitive to dietary conjugated linoleic acid treatment in mice PLoS One 2010 5 e13005 10.1371/journal.pone.0013005 20886002 PMC2946340

[B48] Liu Y Yang L Conde-Knape K Beher D Shearman MS Shachter NS Fatty acids increase presenilin-1 levels and gamma-secretase activity in PSwt-1 cells J Lipid Res 2004 45 2368 2376 10.1194/jlr.M400317-JLR200 15375184

[B49] Wang WX Wilfred BR Madathil SK Tang G Hu Y Dimayuga J Stromberg AJ Huang Q Saatman KE Nelson PT miR-107 regulates granulin/progranulin with implications for traumatic brain injury and neurodegenerative disease Am J Pathol 2010 177 334 345 10.2353/ajpath.2010.091202 20489155 PMC2893676

[B50] Xie H Lim B Lodish HF MicroRNAs induced during adipogenesis that accelerate fat cell development are downregulated in obesity Diabetes 2009 58 1050 1057 10.2337/db08-1299 19188425 PMC2671055

[B51] Liao Y Lönnerdal B Global microRNA characterization reveals that miR-103 is involved in IGF-1 stimulated mouse intestinal cell proliferation PLoS One 2010 5 e12976 10.1371/journal.pone.0012976 20886090 PMC2944884

[B52] Dobashi Y Kudoh T Matsumine A Toyoshima K Akiyama T Constitutive overexpression of CDK2 inhibits neuronal differentiation of rat pheochromocytoma PC12 cells J Biol Chem 1995 270 23031 23037 10.1074/jbc.270.39.23031 7559442

[B53] Wines-Samuelson M Handler M Shen J Role of presenilin-1 in cortical lamination and survival of Cajal-Retzius neurons Dev Biol 2005 277 332 346 10.1016/j.ydbio.2004.09.024 15617678

[B54] Puzzo D Vitolo O Trinchese F Jacob JP Palmeri A Arancio O Amyloid-beta peptide inhibits activation of the nitric oxide/cGMP/cAMP-responsive element-binding protein pathway during hippocampal synaptic plasticity J Neurosci 2005 25 6887 6897 10.1523/JNEUROSCI.5291-04.2005 16033898 PMC6725343

[B55] Garzon R Pichiorri F Palumbo T Visentini M Aqeilan R Cimmino A Wang H Sun H Volinia S Alder H Calin GA Liu CG Andreeff M Croce CM MicroRNA gene expression during retinoic acid-induced differentiation of human acute promyelocytic leukemia Oncogene 2007 26 4148 4157 10.1038/sj.onc.1210186 17260024

[B56] Das Neves L Duchala CS Godinho F Haxhiu MA Colmenares C Macklin WB Campbell CE Butz KG Gronostajski RM Disruption of the murine nuclear factor I-A gene (Nfia) results in perinatal lethality, hydrocephalus, and agenesis of the corpus callosum Proc Natl Acad Sci 1999 96 11946 11951 10.1073/pnas.96.21.11946 10518556 PMC18392

[B57] Teipel SJ Bayer W Alexander GE Zebuhr Y Teichberg D Kulic L Schapiro MB Moller H-J Rapoport SI Hampel H Progression of corpus callosum atrophy in Alzheimer disease Arch Neurol 2002 59 243 248 10.1001/archneur.59.2.243 11843695

[B58] Yamakuchi M Lotterman CD Bao C Hruban RH Karim B Mendell JT Huso D Lowenstein CJ P53-induced microRNA-107 inhibits HIF-1 and tumor angiogenesis Proc Natl Acad Sci 2010 107 6334 6339 10.1073/pnas.0911082107 20308559 PMC2851979

[B59] Del Bo R Scarlato M Ghezzi S Martinelli Boneschi F Fenoglio C Galbiati S Virgilio R Galimberti D Galimberti G Crimi M Ferrarese C Scarpini E Bresolin N Comi GP Vascular endothelial growth factor gene variability is associated with increased risk for AD Ann Neurol 2005 57 373 380 10.1002/ana.20390 15732116

[B60] Bai S Nasser MW Wang B Hsu SH Datta J Kutay H Yadav A Nuovo G Kumar P Ghoshal K MicroRNA-122 inhibits tumorigenic properties of pepatocellular carcinoma cells and sensitizes these cells to sorafenib J Biol Chem 2009 284 32015 32027 10.1074/jbc.M109.016774 19726678 PMC2797273

